# Streamlining search methods to update evidence and gap maps: A case study using intergenerational interventions

**DOI:** 10.1002/cl2.1380

**Published:** 2024-01-07

**Authors:** Morwenna Rogers, Anthea Sutton, Fiona Campbell, Rebecca Whear, Alison Bethel, Jo Thompson Coon

**Affiliations:** ^1^ Evidence Synthesis Team, NIHR ARC South West Peninsula (PenARC) University of Exeter Medical School Exeter UK; ^2^ SCHARR, University of Sheffield, Regent Court Sheffield UK; ^3^ Population Health Sciences Institute Newcastle University Newcastle UK

**Keywords:** databases, evidence and gap maps, search methods, search summary tables, update searching

## Abstract

**Background:**

Evidence and Gap Maps (EGMs) should be regularly updated. Running update searches to find new studies for EGMs can be a time‐consuming process. Search Summary Tables (SSTs) can help streamline searches by identifying which resources were most lucrative for identifying relevant articles, and which were redundant. The aim of this study was to use an SST to streamline search methods for an EGM of studies about intergenerational activities.

**Methods:**

To produce the EGM, 15 databases were searched. 8638 records were screened and 500 studies were included in the final EGM. Using an SST, we determined which databases and search methods were the most efficient in terms of sensitivity and specificity for finding the included studies. We also investigated whether any database performed particularly well for returning particular study types. For the best performing databases we analysed the search terms used to streamline the strategies.

**Results:**

No single database returned all of the studies included in the EGM. Out of 500 studies PsycINFO returned 40% (*n* = 202), CINAHL 39% (*n* = 194), Ageline 25% (*n* = 174), MEDLINE 23% (*n* = 117), ERIC 20% (*n* = 100) and Embase 19% (*n* = 98). HMIC database and Conference Proceedings Citation Index‐Science via Web of Science returned no studies that were included in the EGM. ProQuest Dissertations & Theses (PQDT) returned the highest number of unique studies (*n* = 42), followed by ERIC (*n* = 33) and Ageline (*n* = 29). Ageline returned the most randomised controlled trials (42%) followed by CINAHL (34%), MEDLINE (29%) and CENTRAL (29%). CINAHL, Ageline, MEDLINE and PsycINFO performed the best for locating systematic reviews. (62%, 46% and 42% respectively). CINAHL, PsycINFO and Ageline performed best for qualitative studies (41%, 40% and 34%). The Journal of Intergenerational Relationships returned more included studies than any other journal (16%). No combinations of search terms were found to be better in terms of balancing specificity and sensitivity than the original search strategies. However, strategies could be reduced considerably in terms of length without losing key, unique studies.

**Conclusion:**

Using SSTs we have developed a method for streamlining update searches for an EGM about intergenerational activities. For future updates we recommend that MEDLINE, PsycINFO, ERIC, Ageline, CINAHL and PQDT are searched. These searches should be supplemented by hand‐searching the Journal of Intergenerational Relationships and carrying out backwards citation chasing on new systematic reviews. Using SSTs to analyse database efficiency could be a useful method to help streamline search updates for other EGMs.

## 1 BACKGROUND

In 2022 researchers from the Universities of Exeter and Sheffield produced an evidence and gap map (EGM) for the Campbell Collaboration (Campbell et al., 2023) to examine the effectiveness of intergenerational activities for young people and older adults, and the experiences of those who took part in them. The EGM included systematic reviews, randomised controlled trials (RCTs), other comparative studies and qualitative research.

EGMs provide an overview and a visual representation to the research evidence for a specific topic area. If kept up to date they are a key tool in decision making, providing links to the latest research and provide a useful starting point for researchers in identifying where there may be gaps in the research (Snilstveit et al., 2016). Guidance produced by the Campbell Collaboration recommends that they are updated annually (White et al., 2020).

Intergenerational programmes are activities designed to bring younger and older people together, such as buddy systems that put older people with life experience and skills alongside younger people as a way of promoting positive health, social or educational outcomes, for individuals and for communities. The interventions vary in format and are delivered in a diverse range of settings.

A comprehensive search is a crucial but time‐consuming part of any type of systematic review, including EGMs. To ensure that all relevant articles are retrieved, a range of databases are usually searched, using a wide selection of search terms and controlled vocabulary for each one. For the intergenerational intervention EGM, the search written for the database MEDLINE (via Ovid) alone contained 77 lines of search strings, and this search was translated for another 16 databases. Before a systematic review is published it is recommended that database searches be updated so that the review contains the most current literature (Lefebvre et al., 2022). Search Summary Tables (SSTs) can be helpful in identifying which resources were the most fruitful (Bethel et al., 2021). At the end of the review process, information is collated including the databases searched, the number of results returned by each one, the number of included records found on each one, and whether any records were found subsequently by supplementary searches that were not identified by the initial searches. This information can then be used to streamline searches for future updates and related reviews saving time spent searching redundant databases, restricting the search terms used to the most effective, and reducing unnecessary screening burden (Miwa et al., 2014).

Searching for social science literature is challenging enough because it is diverse, located across a range of media types including journals, books, practitioner publications, theses, and reports from government bodies, charities, or other organisations (Papaioannou et al., 2010). Intergenerational research in particular is time‐consuming to search for because the topic crosses different disciplines (e.g., health, mental health, wellbeing, education and social care) and populations (children and older adults). It is likely therefore that relevant articles will be located across a variety of databases. Social science databases are not as straightforward to search as medical databases in medical disciplines. The terminology is more ambiguous. Indexing and use of controlled vocabulary is not as rigorous as it is for large medical databases such as MEDLINE (McGinn et al., 2014; Papaioannou et al., 2010). Furthermore, social science databases are more likely to be hosted by platforms that are not designed for systematic searching (Bethel & Rogers, 2014). A recent study advocates for a more tailored approach to searching in complex systematic reviews (Cooper et al., 2022).

Iterative search methods to improve efficiencies of systematic reviews have been investigated before. Using a sample of systematic reviews and their included studies, Bramer et al. (2017) showed that a combination of Embase, MEDLINE, Web of Science Core Collection and Google Scholar performed best achieving 100% recall in 72% of systematic reviews across a range of topics and study designs.

Database searches alone are not usually sufficient to locate all the relevant literature for any given topic (Cooper et al., 2017). This might be because relevant articles are not indexed on the databases searched, or the articles do not include abstracts, or the keywords used in the search are insufficient. Guidelines for systematic searching recommend that a variety of search methods be used, not limited to database searching (Lefebvre et al., 2022). These methods include citation searching (e.g., using the reference lists of key papers), searching websites, scrolling through journal contents or contacting authors. These supplementary search methods are particularly important in retrieving grey literature, that is, literature not published in a conventional way. Dissertations and standalone reports are examples of grey literature.

To streamline the process of updating the EGM, and to improve the efficiency of future reviews that investigate intergenerational interventions, we investigated the utility of different resources and search techniques for this specific topic area.

Therefore, the aim of this study was to identify which resources and search terms were the most useful in retrieving articles about intergenerational activities. Specifically, the research questions were:

1. Which databases store research evidence about intergenerational activities?
2. What supplementary search methods perform best for returning research literature about intergenerational activities?
3. Where was grey (unpublished) literature located?
4. Which databases performed best in terms of sensitivity and specificity?
5. How did the resource perform in terms of locating the different types of study?
6. Which search terms and subject headings were the most powerful for finding relevant research on intergenerational activities?

## 2 METHODS

As part of the EGM project, information specialists (AS and MR) searched MEDLINE (via Ovid), Embase (via Ovid), PsycINFO (via Ovid), CINAHL (via EBSCOHost), Social Policy and Practice (via Ovid), Health Management Information Consortium (via Ovid), Ageline (via EBSCOhost), ASSIA (via ProQuest), Social Science Citations Index (via Web of Science), ERIC (via EBSCOhost), ProQuest Dissertations and Theses, Community Care Inform Children, Research in Practice for Children, Conference Proceedings Citation Index‐Science (CPCI‐S) (via Web of Science), the Campbell Library, the Cochrane Database of Systematic Reviews (Cochrane Library) and the CENTRAL database (Cochrane Library) between 22nd and 30th July 2021, using terms for intergenerational activities, older adults, young people, and locations such as care homes and schools where these activities might be carried out. Although the search was developed for MEDLINE, it was tailored for the other databases in terms of relevant subject headings and concepts. The full search strategies for the EGM are available via the review (Campbell et al., 2023).

Grey literature was sought via relevant organisations websites: Age UK, Age International, the Centre for Ageing Better, Barnado's, Children's Commission, UNICEF, Generations Working Together, the Intergenerational Foundation, Linking Generations and The Beth Johnson Foundation, and the Ottawa initiative called Older Adults and Students for Intergenerational support (OASIS https://www.oasis-aesi.com/) between 28th January 2022 and 2nd February 2022 by either examining the resources section of the website or entering ‘intergenerational’ into the search box.

Backwards citation chasing (checking the included studies and reference lists) was carried out on the included systematic reviews to identify any RCTs and other systematic reviews not already included in the EGM. The contents of the Journal of Intergenerational Relationships were hand‐searched.

All results were downloaded into EndNote X9 (Clarivate). A record was made of the database source for all articles before deduplication.

An SST was completed for the 500 records finally included in the EGM. Data captured included the study type (RCT, observational study, Non RCT, qualitative, systematic review or mixed) and the databases the articles were located on. The full SST for all 500 records can be seen in Supporting Information: Appendix 1.

The SST was used to establish:

1. The number of records from the EGM that were found on each of the 15 databases searched.
2. The number of unique articles held by each database, that is, those that were not found by searching any of the other 16 databases.
3. Which databases returned no unique articles, that is, those that could be found by searching other databases.
4. Which databases performed best in terms of sensitivity (i.e., those which returned the most included articles), and specificity (those which returned the least amount of irrelevant literature).
  Sensitivity and specificity were calculated as follows:
Sensitivity=Numberofincludedrecordsindatabase/NumberofincludedrecordsinEGM,
Specificity=Numberofincludedrecordsindatabase/Totalrecall.
5. Whether any databases performed better for returning specific study types.

Data collected during the production of the EGM were used to find the following:

1. Which journals contained the most relevant articles and could therefore be deemed most useful to hand‐search for intergenerational research.
2. Which search terms and combinations of terms were the most sensitive (i.e., returned the most records from the test set) and the most specific (i.e., returned a high proportion of included records to irrelevant records) for the best performing databases.

The aim of this work was not to produce database search filters for records about intergenerational activities, which would not be possible without an independently generated test set of articles. Rather, we aimed to establish if our own searches could be made more efficient for future updates of the EGM, by using the most powerful search terms and combinations of terms and removing those which were found to be redundant.

When completing a SST, it is good practice to check whether studies are included on databases even if not returned by the search strategies (Bethel et al., 2021). However, since we had 500 references, time and resource constraints meant that this stage was not possible.

### 2.1 Assessing search terms

The original search strategies were rerun in Ageline, CINAHL, MEDLINE, ERIC and PsycINFO, which were the databases that returned most high quality or unique articles. Embase was not selected for this process. This was because it returned no unique articles of high quality, and five of the six unique articles it returned were conference abstracts. Terms used in the database searches were tested against the records included in the EGM that were returned by those databases. To form the test set for each database, the individual included records had to be retrieved in isolation and then combined with an ‘OR’. Where it was not possible to isolate records (e.g., where duplicates or similar records with authors and title words in common were returned with the included study) that study was removed from the test. Sensitivity and specificity calculations were recorded for search strings and combinations.

## 3 RESULTS

For the EGM, over 12,000 articles were retrieved from 17 databases, key websites and through citation chasing, with 8638 records being screened resulting in 500 eventually included in the EGM.

### 3.1 Value of databases

Two databases, CPCI‐S and HMIC returned none of the articles included in the EGM. Table 1 shows the number of included articles that were found on the different databases searched.

**Table 1:** Number of included articles found on different databases searched.

Database	Number of included articles (%)
APA PsycINFO via Ovid	202 (40)
CINAHL via EBSCOhost	194 (39)
AGELINE via EBSCOhost	174 (35)
MEDLINE via Ovid	117 (23)
ERIC via EBSCOhost	100 (20)
Embase via Ovid	98 (20)
SSCI (Social Science Citation Index) via Web of Science	79 (16)
PQDT (ProQuest Dissertations and Theses) via ProQuest	71 (14)
SPP (Social Policy and Practice) via Ovid	68 (14)
ASSIA via ProQuest	41 (8)
CENTRAL via the Cochrane Library	22 (4)
CPCI‐S (Conference Proceedings Citation Index‐Science)via Web of Science	0
HMIC (Health Management Information Consortium) via Ovid	0

No single database returned all the included articles, based on the searches used for the EGM. Table 2 shows the number of articles unique to individual databases (i.e., those that were not found on any other database with our strategies).

**Table 2:** Number of articles unique to individual databases.

Database	Number of unique articles
PQDT	42
ERIC	33
Ageline	29
PsycINFO	19
CINAHL	17
SPP	14
MEDLINE	11
SSCI	10
Embase	6
ASSIA	2
CENTRAL	1
CPCI‐S	0
HMIC	0

### 3.2 Value of handsearching journals

The journals that appeared most frequently in the EGM were the Journal of Intergenerational Relationships (82 articles included in the EGM, including 10 systematic reviews) and Educational Gerontology (68 articles). Other than these two, only five titles, Activities, Adaptation & Aging (6 articles), Gerontologist (Dorgo et al., 2011), Gerontology & Geriatrics Education (Reinsch & Tobis, 1991), Journal of Applied Gerontology (Miwa et al., 2014) and Journal of Gerontological Social Work (McGinn et al., 2014) provided more than five included articles.

### 3.3 Value of citation chasing

Ten additional articles were found by carrying out backwards citation searching on included systematic reviews. These included one further systematic review (Chonody, 2015) and four additional RCTs (Dorgo et al., 2011, 2013; Tan et al., 2006; Varma et al., 2016).

### 3.4 Database performance

Table 3 shows the sensitivity and specificity for all databases that returned included articles.

**Table 3:** Sensitivity and specificity for all databases that returned included articles.

Database	No. of relevant articles retrieved	Total no. of articles retrieved	Sensitivity (No. of relevant articles retrieved/500)	Specificity
PsycINFO	202	1307	0.40	0.15
CINAHL	194	1969	0.39	0.1
Ageline	174	918	0.35	0.19
MEDLINE	117	1567	0.23	0.07
ERIC	100	1241	0.20	0.08
Embase	98	707	0.20	0.14
SSCI	79	254	0.16	0.31
PQDT	71	1334	0.14	0.05
SPP	68	538	0.14	0.12
ASSIA	41	1947	0.08	0.02
CENTRAL	22	248	0.04	0.09

### 3.5 RCTs

38 RCTs were included in the EGM. The database Ageline returned the most RCTs (Tan et al., 2006), followed by CINAHL (Chonody, 2015) and then MEDLINE (Cooper et al., 2022). Only 10 of the included RCTs were retrieved from the CENTRAL database. 20 RCTs included in the EGM were not on the CENTRAL database, 3 of which were dissertations. One absent RCT was only added to CENTRAL after the date of the search. Another three were present on CENTRAL but were not retrieved by the search. In total, CENTRAL returned 28 included records, but 11 of these were judged not to be RCTs during coding of the articles.

Several databases (ERIC, CENTRAL, Ageline, PQDT and APA PsycINFO) returned unique RCTs that were not captured by searching any other database (Tables 4).

**Table 4:** Number of studies found on databases by study type.

Database	No. of RCTs	No. of systematic reviews	Qualitative studies
Ageline	16	12	46
CINAHL	13	16	55
MEDLINE	10	11	32
CENTRAL	10	0	2
PsycINFO	9	11	54
Embase	7	8	21
PQDT	7	2	19
ASSIA	4	3	10
ERIC	3	0	29
SPP	2	7	18
SSCI	1	6	17

### 3.6 Systematic reviews

26 systematic reviews were included in the EGM. 16 were found on CINAHL, 12 on Ageline and 11 on both MEDLINE and APA PsycINFO. Unique systematic reviews were found on Ageline (1 review), CINAHL (1 review), Social Policy and Practice (2 reviews), MEDLINE (1 review) and PQDT (2 reviews). One review was only found by backwards citation searching.

### 3.7 Qualitative studies

134 articles reporting qualitative studies (excluding mixed methods studies) were included in the EGM. 55 were found on CINAHL, 54 on PsycINFO and 46 on Ageline. Unique articles reporting qualitative studies were found on PQDT (12), ERIC (10), Social Policy and Practice (2), Social Science Citation Index (4), Ageline (5), PsycINFO (3), CINAHL (6), MEDLINE (3), Embase (1) and ASSIA (2).

### 3.8 Performance of search terms

Search terms were tested on five databases: MEDLINE (via Ovid), CINAHL (via EBSCOhost), Ageline (via EBSCOhost), ERIC (via EBSCOhost) and APA PsycINFO (via Ovid).

#### 3.8.1 MEDLINE

**Figure 1:**
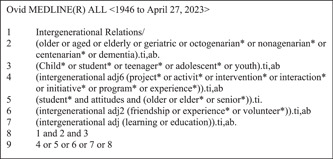
Streamlined MEDLINE search.

Search terms were tested on 28th April 2023. The original search returned 1936 results during testing. 109 included records were used as the test set on MEDLINE. No combinations of terms improved sensitivity of the search. However, a search strategy that was nine lines long and returned 1779 results only missed three of the included hits (Table 5). One (Chippendale & Boltz, 2015) would have been picked up by other databases. The remaining two however were only found on MEDLINE. One was an observational study from 1991 (Reinsch & Tobis, 1991), the other a qualitative study from 2009 (McCalman et al., 2009). Figure 1 shows the streamlined search for MEDLINE.

**Table 5:** Search term testing results on MEDLINE.

	No. of strings (lines of search)	No. of records returned	No. of included records returned
Search 1 (Original)	77	1936	109
Search 2 (Streamlined)	9	1779	106

The original search produced for the EGM can be seen in Supporting Information: Appendix 2.

#### 3.8.2 CINAHL

191 included records were located on CINAHL at the time of testing. No combinations of terms were found to be more effective than the original strategy in terms of balancing sensitivity and specificity. Table 6 shows the comparison between the original search on CINAHL and the results of our best fit streamlined strategy.

**Table 6:** Search terms testing on CINAHL.

	No. of strings (lines of search)	No. of records returned	No. of included records returned
Search 1 (Original)	135	1969	194/194
Search 2 (Streamlined)	6	1809	186/191

Of the five included records that were missing from the streamlined search, one had no abstract, one was a conference abstract, one was a survey from 2013 and one was a dissertation about quality of life in nursing homes more generally. There was one RCT (Leung et al., 2012) that was not returned with the streamlined strategy about service training for medical students to improve attitudes towards older adults. This trial was not however unique to CINAHL.

Table 7 shows the streamlined strategy for CINAHL.

**Table 7:** Streamlined strategy for CINAHL.

#	Query	Results
S8	S5 OR S7	1751
S7	S1 AND S6	185
S6	TI ((student* or young or children*) and (old* or ageing or aging or elder*) and (attitude* or perception*)) OR AB ((student* or young or children*) and (old* or ageing or aging or elder*) and (attitude* or perception*))	5916
S5	S3 OR S4	1708
S4	TI (intergenerational N3 (program* or activit* or intervention* or interaction* or engag* or relationship* or mentor* or connect* or class* or exercis* or project* or learning or opportunit* or volunteer* or experienc* or group* or exchange* or perception* or model* or alliance* or forum or event* or initiative*))) OR AB (intergenerational N3 (program* or activit* or intervention* or interaction* or engag* or relationship* or mentor* or connect* or class* or exercis* or project* or learning or opportunit* or volunteer* or experienc* or group* or exchange* or perception* or model* or alliance* or forum or event* or initiative*))	1604
S3	S1 AND S2	211
S2	TI (child* or young or student* or teenager* or youth*) and (elder* or old* or resident*)	8514
S1	(MM ‘Intergenerational Relations’)	4054

#### 3.8.3 Ageline

168 included records were isolated individually on Ageline at the time of testing. No combinations of terms were found to be more effective that the original strategy in terms of balancing sensitivity and specificity. Although there were more lines of search in the streamlined strategy, these lines contained far fewer phrases. Table 8 shows the comparison between the original search on Ageline and the results of our best fit streamlined strategy.

**Table 8:** Original Ageline search results compared with streamlined strategy.

	No. of strings (lines of search)	No. of records returned	No. of included records returned
Search 1 (Original)		918	174/174
Search 2 (Streamlined)	5	1136	167/168

The one record missed by the streamlined search was a mixed methods study that was found in three other databases (Vélez Ortiz et al., 2012). The remaining six were either not found or easily isolated at the time of testing on Ageline.

Table 9 shows the streamlined strategy for Ageline.

**Table 9:** Streamlined strategy for Ageline.

	Search terms	Results
S7	S1 OR S2 OR S3 OR S4 OR S5 OR S6	1136
S6	TI Intergenerational Volunteering OR AB Intergenerational Volunteering	18
S5	TI Generations W3 together OR AB Generations W3 together	76
S4	TI volunteer* W5 school* OR AB volunteer* W5 school*	61
S3	TI intergenerational project OR AB intergenerational project*	167
S2	TI intergenerational program* OR AB intergenerational program*	697
S1	DE ‘Intergenerational Programs’	638

#### 3.8.4 PsycINFO

199 included records were isolated individually for testing on PsycINFO in June 2023. No combinations of terms were found to be more effective than the original searches in terms of balancing sensitivity and specificity. A streamlined strategy of one line (intergenerational.ti) retrieved 180 included records but returned over 3000 more records in total. Attempts to create a streamlined strategy using the best search terms resulted in a suboptimal balance of sensitivity and specificity (Table 10).

**Table 10:** Original PsycINFO search results compared with streamlined strategy.

	No. of strings (lines of search)	No. of records returned	No. of included records returned
Search 1 (Original)	77	1307	202/202
Search 2 (Streamlined)	1	4673	180/199

The single term search missed 19 records from the original search. Of these, 6 were found by searching PsycINFO. Four were published between 1984 and 1986, 1 was a book chapter from 2019, and one was a mixed methods study (Brant & Studebaker, 2021) from 2021 published in the Journal of Intergenerational Relationships.

#### 3.8.5 ERIC

All 100 included records from ERIC were isolated for testing. No combinations of terms were found to be more effective than the original searches in terms of balancing sensitivity and specificity. One streamlined strategy retrieved the 100 records. A second retrieved 98 but with over 600 fewer records returned. Of the two records missed, one was a observational study from 1991 that was unique to ERIC (Petri et al., 2001) and the other was a report from 1988 that was also found in Ageline (Freedman, 1988). Search 3 was the same as search 2 except with the inclusion of the subject heading for Intergenerational Programs (i.e., this subject heading returned an additional 635 records to retrieve two).

Table 11 shows the comparison between the original strategy and the results when the terms were tested and Table 12 shows the streamlined search strategy for ERIC.

**Table 11:** Original ERIC search results compared with streamlined strategies.

	No. of strings (lines of search)	No. of records returned	No. of included records returned
Search 1 (Original)	4	1241	100/100
Search 2 (Streamlined sensitive)	4	2130	100/100
Search 3 (Streamlined precise)	3	1495	98/100

**Table 12:** Streamlined search strategy for ERIC.

	Search terms	Results
S4	S1 OR S2 OR S3	2130
S3	DE ‘Intergenerational Programs’	1149
S2	TI ((volunteer* or ‘voluntary’) N5 (‘old aged’ or elderly or ‘geriatric’ or pensioner* or veteran* or older)) OR AB ((volunteer* or ‘voluntary’) N5 (‘old aged’ or elderly or ‘geriatric’ or pensioner* or veteran* or older))	370
S1	TI Intergenerational	1087

## 4 DISCUSSION

To remain current, EGMs should be updated regularly (White et al., 2020). The use of automation software helps with review tasks such as screening however at present there is no software capable of running multiple update searches across a range of databases and platforms without human input. In addition, there is little guidance on how to update systematic searches, beyond advised date limits and the practicalities of how to manage the references (Bramer & Bain, 2017; Moher et al., 2007).

In general, when EGMs or other reviews are updated, the original strategies are used without any knowledge of how individual terms and phrases performed, resulting in an additional screening load with potentially little gain. This is a time‐consuming process, particularly if the update searches are not being carried out by the person who ran the original searches, or if the platforms used have an insufficient function for saving and rerunning multiple lines of search.

As our topic (intergenerational interventions) was very broad in terms of covering both health and social science, with populations from two distinct groups (young people and older adults), and with all comparative, qualitative and review study types included, it was deemed necessary to search across a large number of databases.

To allow us the potential of updating the EGM in the future we wanted to identify the most efficient ways of searching for relevant articles to avoid searching multiple databases with many search terms for no added benefit.

It is understood that searching for social science articles is problematic due to the diverse nature of topics. Our findings support this view, showing that articles included in our EGM were located across a broad range of databases, covering medicine, psychology, nursing and education.

It should be noted that decisions about which databases to test and use in an update were based on both the evidence supplied by the SST and the experience and knowledge of the team in search methods and databases. For example, the decision to omit Embase from future updates was based on the quality of the studies returned and the number of unique articles, as well as knowledge of its coverage, which tends towards biomedical research rather than social sciences.

In terms of databases, most performed well with almost all returning unique articles that were not retrieved from any other databases. Only HMIC and CPCI‐S returned no unique articles, meaning that for future updates, it would be worth considering the omission of these databases.

The best performing databases for this topic were MEDLINE, Ageline, PsycINFO, CINAHL and ERIC. These five databases should be searched as a minimum for any updates to the EGM, or any reviews into intergenerational activities. Although Embase returned six unique articles, five of these were conference abstracts, and none were RCTs, systematic reviews or articles describing high‐quality qualitative research. Ideally Embase would also be searched as part of a systematic review however if time and resources are constraints, the omission of this database would be more justifiable than that of the others.

In terms of RCTs, the inclusion of CENTRAL was found to be less conclusive, returning only one unique RCT and only returning nine in total. This was surprising, given that CENTRAL is a database of RCTs, populated through regular searches of much larger databases. Also surprising was that several of the included articles returned by CENTRAL were deemed by our review team not to be RCTs. The best performing databases for returning RCTs were Ageline, CINAHL and MEDLINE. Embase only returned 7 of the 38 RCTs. However, this finding may reflect the nature of the topic, covering social science, meaning that more articles were likely to be found on these databases regardless.

CINAHL, PsycINFO and Ageline were the most useful databases for finding qualitative research, reflecting previous research in this area (Rogers et al., 2018).

A surprising number of included articles were found in dissertations, meaning that ProQuest Dissertations & Theses (PQDT) was an essential resource and should also be searched in future updates. Previous research by Hartling et al. (2017) found that dissertations represented a small percentage of included articles in a selection of Cochrane reviews, and their inclusion had negligible impact on the results. In fact, their inclusion could lead to an over‐estimation of effectiveness. However, these reviews focused on RCTs for medical topics whereas our EGM contained additional study types and covered social science as well as medicine. It is worth noting that for our EGM, PQDT returned seven RCTs, one of which was only retrieved by our search on PQTD. Although this investigation was specific to intergenerational interventions, it might be inadvisable for similar broad topics to exclude dissertations from review searches.

In the original searches we hand‐searched only one journal, the Journal of Intergenerational Relationships. Although no new relevant studies were found, the fact that 82 included articles, including 4 RCTs and 10 systematic reviews came from this journal means that for any future update, it would be advisable to hand‐search its contents as a supplementary search.

Backwards citation searches of systematic reviews proved to be lucrative in this project, returning 11 additional articles for the EGM, including 1 systematic review and 4 RCTs. For future updates, it would be advisable to check the included studies within any newly identified systematic review.

We searched a number of websites for additional grey literature and although we found documents relating to intergenerational activities we did not find any additional articles for inclusion in our AGM.

With regard to search strategies, for MEDLINE, ERIC, Ageline, PsycINFO and CINAHL we were unable to develop strategies that performed better than the originals in terms of sensitivity. This is perhaps unsurprising giving that we only used one test of papers – those that we had found with this strategy. The aim of this work was not to develop a search filter for intergenerational research but to streamline future update searches of this EGM. We were also unable to improve much on specificity. However, we were able to cut down significantly on the individual lines of search used for each database which could translate as a time saving for the researcher performing the search. It is also worth mentioning that the original search strategies were designed by two experienced information specialists with consultation across a team of experienced reviewers, and therefore the search was originally designed and tested extensively with both sensitivity and specificity in mind.

Some of our time‐saving searches returned more articles than the original searches, which would mean that although the searches would be more efficient for the searcher, the screening load would increase. For example, the single search *intergenerational.ti* in PsycINFO returned all but 19 of the 199 articles searched for within it. Of those that were unique to PsycINFO, 4 were published pre‐1990, 1 was a book chapter and 1 was published in the Journal of Intergenerational Relationships, which should be hand‐searched in future updates. As a date limitation would be used for future updates thus reducing the number of articles returned, this single line search for PsycINFO might be worth considering.

### 4.1 Limitations

A clear limitation is that search strategies were being tested against a single set of articles. Rather than designing a filter for intergenerational studies, we aimed to examine which search terms and combinations of search terms performed the best, and which were redundant. In addition, when examining streamlined search strategies we compared the results to the original strategies. Where we found that articles were ‘lost’ by the new strategies we checked if they were caught by other databases using the SST which was also based on the original searches, that is, new streamlined search results were not compared with each other across databases.

Where we describe records as being ‘unique’ to a particular database, we mean that we only found that record on a single database. Due to the high number of articles, it was not feasible to check whether these unique articles were in fact on other databases but had been missed by the searches we ran on them.

## 5 CONCLUSION

Using an SST, we have produced a method for updating the EGM that does not require a complete repetition of the original search methods.

If future searches for articles additional to those in the existing EGM are required, researchers should search as a minimum MEDLINE, Ageline, PsycINFO, CINAHL and ERIC. Ideally the searches as previously developed for the EGM should be used to ensure the optimum balance of sensitivity and specificity, but if this is not practical due to limitations in expertise or time, the streamlined searches could be considered. PQDT should also be considered if a broad range of study types are required. The Journal of Intergenerational Relationships should be hand‐searched, and reference lists of any new systematic reviews should also be checked for additional primary articles.

This research, although specific to intergenerational activities, gives an example of how SSTs could help to streamline annual updates of future EGMs. EGMs should be regularly updated to keep them current and relevant. Researchers producing EGMs should consider the update process from the outset and might wish to include the production of an SST along with methods for future updates in the EGM protocol.

## DECLARATIONS OF INTEREST

The authors declare no conflicts of interest.

## SOURCES OF SUPPORT

**Internal Sources**

None.

**External Sources**

This article presents independent research funded by the National Institute for Health Research (NIHR) Applied Research Collaboration (ARC) South West Peninsula. The views expressed in this publication are those of the author(s) and not necessarily those of the National Health Service, the NIHR or the Department of Health and Social Care. The funders had no role in study design, data collection and analysis, decision to publish, or preparation of the manuscript.

### PEER REVIEW

The peer review history for this article is available at https://www.webofscience.com/api/gateway/wos/peer-review/10.1002/cl2.1380.

## Supporting information


**Appendix 1**.

Supporting information.

## Data Availability

The data that supports the findings of this study are available in the supplementary material of this article.
