# Supplementary material for: Streamlining search methods to update evidence and gap maps: A case study using intergenerational interventions
Source: Campbell Syst Rev. 2024 Jan 7;20(1):e1380. doi: 10.1002/cl2.1380 (PMC10771710; doi:10.1002/cl2.1380)
Supplement: Supplementary file 2 — Supporting information. [file CL2-20-e1380-s001.docx]

Appendix 2

IGen EGM Medline search strategy

MEDLINE via OvidSp

Database: Ovid MEDLINE(R) ALL <1946 to July 21, 2021>

Search Strategy:

--------------------------------------------------------------------------------

1 Intergenerational Relations/ (4146)

2 (intergenerational or inter generational).ti,ab. (6528)

3 cross age.ti,ab. (109)

4 across generation*.ti,ab. (2061)

5 cross generation*.ti,ab. (438)

6 ((generations or different age groups or all ages or all age groups or mixed ages or mixed age groups or (old* adj2 young*)) adj5 (together or social engagement or connecting)).ti,ab. (257)

7 (intergenerational adj2 (program* or intervention*)).ti. (71)

8 or/1-6 (11703)

9 adolescent/ or child/ or child, preschool/ (3143466)

10 child*.ti,ab. (1463735)

11 (young adj (person or people or male* or female*)).ti,ab. (54389)

12 (youth* or teen*).ti,ab. (110196)

13 young offender*.ti,ab. (499)

14 (school and pupil*).ti,ab. (4871)

15 preschooler*.ti,ab. (7441)

16 student*.ti,ab. (304578)

17 (girl or girls or boy or boys).ti,ab. (240923)

18 or/9-17 (3888702)

19 exp Aged/ (3276163)

20 dementia.ti,ab. (115820)

21 alzheimer*.ti,ab. (156770)

22 old*.ti,ab. (1566913)

23 elderly.ti,ab. (258523)

24 geriatric.ti,ab. (44411)

25 (residents or resident).ti,ab. (170641)

26 (elder or elders).ti,ab. (16913)

27 (retired or retiree*).ti,ab. (7895)

28 veteran*.ti,ab. (38644)

29 grandfriend*.ti,ab. (0)

30 seniors.ti,ab. (7890)

31 (senior adj citizen*).ti,ab. (1548)

32 (centarian* or centenarian* or nonagenarian* or octagenarian* or octogenarian* or sexagenarian* or septuagenarian*).ti,ab. (6890)

33 or/19-32 (4773325)

34 program*.ti,ab. (937612)

35 activit*.ti,ab. (3230552)

36 interaction*.ti,ab. (1398324)

37 (project or projects).ti,ab. (220965)

38 intervention*.ti,ab. (1099124)

39 initiative*.ti,ab. (95427)

40 scheme.ti,ab. (104838)

41 visit*.ti,ab. (255175)

42 reading.ti,ab. (118535)

43 (play or playing or playtime).ti,ab. (759787)

44 music.ti,ab. (17586)

45 boardgame*.ti,ab. (3)

46 games.ti,ab. (16559)

47 voluntary.ti,ab. (64481)

48 volunteering.ti,ab. (2125)

49 mentor*.ti,ab. (17791)

50 or/34-49 (7060335)

51 Homes for the Aged/ (14400)

52 Nursing Homes/ (36493)

53 care home*.ti,ab. (4517)

54 nursing home*.ti,ab. (31757)

55 residential care.ti,ab. (3647)

56 ((senior or elderly or old) adj day care).ti,ab. (46)

57 ((hospital* or ward) adj3 geriatric*).ti,ab. (3384)

58 community.ti,ab. (529188)

59 (sheltered adj (housing or accommodation)).ti,ab. (257)

60 (retirement adj (home* or village* or complex*)).ti,ab. (476)

61 (abbeyfield or almshouse*).ti,ab. (65)

62 (geriatric adj (institution* or care)).ti,ab. (2026)

63 assisted living.ti,ab. (2324)

64 own home.ti,ab. (1205)

65 (preschool or preschools).ti,ab. (26214)

66 playgroup*.ti,ab. (142)

67 (school or schools or college*).ti,ab. (398572)

68 (nursery or nurseries).ti,ab. (11824)

69 kindergarten*.ti,ab. (7051)

70 play setting*.ti,ab. (90)

71 (child care setting* or childcare setting*).ti,ab. (423)

72 (child* adj2 day care).ti,ab. (1110)

73 or/51-72 (987221)

74 8 and 18 and 33 and 50 (955)

75 8 and 18 and 50 and 73 (809)

76 8 and 33 and 50 and 73 (516)

77 7 or 74 or 75 or 76 (1567)
